# National genotype prevalence and age distribution of human papillomavirus from infection to cervical cancer in Japanese women: a systematic review and meta-analysis protocol

**DOI:** 10.1186/s13643-021-01686-6

**Published:** 2021-05-05

**Authors:** Matthew Palmer, Kota Katanoda, Eiko Saito, Cecilia Acuti Martellucci, Shiori Ostuki, Shuhei Nomura, Erika Ota, Julia M. L. Brotherton, Jane Hocking

**Affiliations:** 1 Division of Cancer Statistics Integration, Center for Cancer Control and Information Services, National Cancer Center, Tokyo, Japan; 2 Melbourne School of Population and Global Health, The University of Melbourne, Melbourne, Australia; 3 Department of Global Health Policy, Graduate School of Medicine, The University of Tokyo, Tokyo, Japan; 4 Division of Prevention, Center for Public Health Sciences, National Cancer Center, Tokyo, Japan; 5 St Luke’s International University, Global Health Nursing, Tokyo, Japan; 6 VCS Population Health, VCS Foundation, East Melbourne, Australia

**Keywords:** HPV, Human papillomavirus, Vaccination, Japan

## Abstract

**Background:**

Despite prophylactic human papillomavirus (HPV) vaccination being a safe, effective and cost-effective public health intervention for the prevention of cervical cancer, the HPV vaccine is not actively recommended or promoted by the Ministry of Health Labour and Welfare in Japan. With already very low levels of cervical screening below 30%, and vaccination levels that are below levels that award any population effect at 0.3% of the eligible population, cervical cancer mortality is higher than other similar high-income countries at 4.4/100,000 (2900) deaths per year in 2015. There is limited population-based or nationally representative data for HPV genotype distribution in Japan, thus making an assessment of the burden of vaccine-preventable cervical cancer difficult. Therefore, this systematic review and meta-analysis aims to determine the HPV genotype prevalence and age distribution of HPV infection in women with a cytological or histological diagnosis of normal through cervical cancer in Japan. We anticipate this information will guide and enhance programme interventions to reduce vaccine-preventable cervical cancer mortality in Japan.

**Methods:**

PubMed, Embase and the Japan Medical Abstract Society Database will be searched from the date of establishment to March 2021 to identify original research articles that report the prevalence of HPV genotypes in Japanese women with normal cervical cytology, low grade, high grade and cancerous cervical lesions. No exclusion criteria relating to language or publication date will be applied. The quality of the studies will be assessed using the Joanna Briggs checklist for prevalence studies. Randomised control trials, cohort studies, cross-sectional and prevalence studies will be considered eligible. Study findings will be combined using a traditional random-effects or fixed-effects meta-analysis to summarise pooled prevalence and 95% confidence intervals depending on heterogeneity. Subgroup analyses and meta-regression will be used to investigate heterogeneity, and sensitivity analyses will be conducted to assess the robustness of the findings.

**Discussion:**

To our knowledge, this is the first systematic review protocol that includes both Japanese and English peer-reviewed articles for the determination of genotype-specific HPV prevalence in cytological or histological confirmed normal cervical specimens, low- and high-grade intraepithelial lesions and cervical cancers by age in Japan. We anticipate this information will guide and enhance programme interventions to reduce vaccine-preventable cervical cancer mortality in Japan.

**Systematic review registration:**

PROSPERO CRD42018117596

**Supplementary Information:**

The online version contains supplementary material available at 10.1186/s13643-021-01686-6.

## Background

Oncogenic HPV types are causal and necessary factors of cervical cancer [1–3]. It has now been shown that prophylactic HPV vaccines are immunogenic and effective against HPV vaccine genotype infections that can otherwise result in precancerous and cancerous lesions, as long as vaccination occurs prior to HPV infection [4–6]. Global evidence shows that HPV vaccination is safe, and that cross-protection against non-vaccine genotypes and herd effect also occur after vaccination [7–11]. In many countries, HPV vaccination programmes as a public health intervention have now been shown through systematic evaluations to be safe, effective and cost-effective methods for the prevention of HPV cervical infection and related disease [12–14]. Paradoxically in Japan, the implementation experience with HPV vaccination has been problematic.

At the time of implementation of the HPV vaccine programme, vaccination coverage for eligible adolescent girls in some prefectures was as high as 80% [15]. In fact, in light of such success, the HPV vaccine was added to the national routine vaccination register in April 2013 and was recommended the vaccine should be made available to all girls between the age of 12 and 16 [16]. However, in response to a series of reported adverse events in June 2013, the Human Papillomavirus Vaccination Programme was partially suspended by the Japanese Ministry of Health, Labour and Welfare (MHLW) [17]. Since then, the MHLW has directed prefectural governments not to actively recommend or promote adolescent HPV vaccination [18, 19].

As a direct result of this suspension, vaccination coverage amongst adolescent girls has dramatically declined to 0.3%, a level that does not award any population benefit [15, 19–22]. At the same time, the cervical screening participation rate is below 30% [23]. Cervical cancer incidence (12.5/100,000) and mortality (2.19/100,000) since 1991 has decreased. However, more recently, the number of cervical cancer cases has risen from 10,520 (10.9/100,000) in 2013 to 11,200 (11.0/100,000) new cases in 2015, and the number of deaths has risen from 2656 (4.1/100,000) to 2900 (4.4/100,000) deaths over the same time period [23]. Currently, municipalities in Japan comprehensively collect population-level cancer screening performance data and report to the MHLW, whilst mortality data is collected by the National Vital Statistics, and incidence and survival data are collected by the prefectural cancer registries and later the national framework of cancer registries. However, there is still limited nationally representative data in Japan assessing the prevalence of HPV infection at the national or subnational level.

Comprehensive studies conducted internationally describe the genotype prevalence of HPV in many countries [21, 22, 24–27]. However, Japan is commonly underrepresented in these studies. The limited nature of HPV genotype prevalence and data is likely to hinder effective advocacy for and planning of primary prevention strategies. To fill this gap, an estimate of the prevalence of HPV infection is essential and can be used to evaluate vaccine impact after reimplantation of the HPV vaccine in the future.

Evidence-based decision-making that is context specific has been key to the successful advocacy for and development of effective HPV vaccination and screening programmes. Therefore, this systematic review and meta-analysis aims to determine the HPV genotype prevalence and age distribution of HPV infection in women with a cytological or histological diagnosis from normal through to cervical cancer for Japanese women residing in Japan, by best utilising existing data.

## Methods

### Research aims

Primary aims:

1. To determine the HPV genotype-specific prevalence in women with a cytological or histological diagnosis of normal, low- and high-grade cervical intraepithelial neoplasia (CIN) and cervical cancer in Japan.
2. To determine the age-specific prevalence of any HPV infection in women with a cytological or histological diagnosis of normal, low- or high-grade cervical intraepithelial neoplasia (CIN) and cervical cancer in Japan.

Secondary aims:

1. To determine the proportion of infections, precancerous lesions and cervical cancers that could be prevented by prophylactic HPV vaccination or are screening detectable in Japan.
2. To determine the prevalence of HPV infection at the national, prefectural and regional levels in women with a cytological or histological diagnosis of normal, low- or high-grade cervical intraepithelial neoplasia (CIN) and a cervical cancer in Japan.

### Protocol and registration

This protocol was developed in line with the Preferred Reporting Items for Systematic reviews and Meta-Analyses guidelines for protocols (PRISMA-P) [28]. The PRISMA-P Checklist for this study is reported in Table S1. In addition, this review protocol has been registered in the International Prospective Register of Systematic Reviews (PROSPERO), with registration number CRD42018117596.

### Search strategy

PubMed, Embase and ICHUSHI (Igaku Chuo Zasshi) will be searched from inception to March 2021. Search terms will include relevant headings and keywords in the title, abstract and text, including human papillomavirus in Japan. ICHUSHI is the domestic database for the Japan Medical Abstracts Society Database. The use of this database requires the development of a Japanese language search strategy.

The search strategy will use the following general terms, expanded and appropriately modified for each database: ‘Japan’ and ‘human papillomavirus’ or ‘HPV’, and ‘cervical cancer’, and ‘genotype’, for ‘normal cytology’, and ‘cervical disease’ or ‘cervical intraepithelial neoplasia’. For this systematic review, there will be no restrictions on the date or language of articles to be reviewed. This search strategy will be constructed and performed with the assistance of a librarian. The search strategy is outlined in Table S2.

The reference lists of identified studies will also be reviewed, evaluated and included if eligible. Grey literature shall also be considered for inclusion if the abstracts contain sufficient information to assess their eligibility. Possible sources of grey literature will include (1) identified authorities of this subject matter, (2) conference papers and (3) government documents and published guidelines. The search strategy will be developed according to Cochran Guidelines in collaboration with a librarian and subject matter expert in both Japanese and English.

### Eligibility criteria

The population of interest for this review are Japanese people with a cervix residing in Japan who were screened at least once regardless of screening interval and stage of diagnosis. Males will be excluded from this study because they do not have a cervix. If identified, transgender men with a cervix will be included in this analysis. There will be no restriction on the age of participants in the studies for inclusion. Studies will be eligible if they are randomised control trials, case-control studies, case series studies, cohort studies or cross-sectional studies. Systematic reviews will not be eligible but their reference lists will be searched to identify any further eligible studies.

In order to achieve comparability to other international studies of HPV genotype prevalence [29], the following inclusion criteria shall be used: (1) studies that assess cervical carcinoma, low-grade or high-grade cervical lesions must include a minimum of 20 cases [21, 22, 24]; (2) studies that describe HPV infection in normal cytology must include a minimum of 100 cases [25–27]; (3) studies must include at least one HPV genotype; (4) DNA or RNA polymerase chain reaction (PCR)-based assays should be used and sufficiently described; and (5) the study must include a detailed methodological description of cervical sampling techniques.

Studies must have been performed in Japan. For studies that were not conducted in Japan, or for multi-country studies, only studies containing primary data reporting HPV genotype prevalence for women resident in Japan will be included. Studies using nucleic acid testing of blood or blood components to detect HPV or reporting HPV prevalence in anatomic sites other than the cervix will be excluded from calculations of prevalence.

### Selection of studies

Covidence review software will be used to screen titles and abstracts of all studies that are initially identified by two independent reviewers according to the selection criteria [30]. The text of all potentially relevant studies will then be evaluated in detail against the eligibility criteria by two independent reviewers.

For the full-text review, the reviewers will independently classify articles as (1) included, (2) excluded or (3) maybe. A maybe status will imply that a decision to include or exclude the article is dependent on additional information being obtained from the author. Where additional information is needed, the corresponding author of the study will be contacted via email. A second email will be sent after 1 week in the event of no response to the initial email. A 2-week waiting period after the submission of the second email will be allowed for sufficient response. After which, these studies will be excluded [31]. Articles that both reviewers classify as excluded will be removed, whereas those that both reviewers classify as included will be included. Discrepancies will be resolved through discussion with a third independent reviewer until consensus is obtained. The opinion of a subject matter expert will be sought, if necessary.

In accordance with the PRISMA guidelines, a summary of the search process, study selection and reasons for exclusion of studies will be included [32]. A summary of all selected studies will also be included.

### Outcome measures

The outcome measure of interest in this study is the HPV genotype-specific prevalence in women with a cytological or histological diagnosis of normal, low- or high-grade lesions or cervical cancers. HPV prevalence will be measured in cervical specimens from women where cytological classification is defined as normal, atypical squamous cells of undetermined significance (ASCUS), low-grade squamous intraepithelial lesion (LSIL) and high-grade squamous intraepithelial lesion (HSIL), and histological classification is defined as normal, cervical intraepithelial neoplasia 1 (CIN1), cervical intraepithelial neoplasia 2 (CIN2), cervical intraepithelial neoplasia 3 (CIN3), adenocarcinoma in situ (AIS), invasive cervical cancer (ICC)—unspecified, ICC—squamous cell carcinoma or ICC—adenocarcinoma.

### Definitions

Type-specific prevalence is defined as the total number of women who are positive for a HPV genotype (*n*), expressed as a proportion of the total number of women who are tested for the given HPV genotype (*N*) with a DNA- or RNA-based PCR assay. This is given by the equation below:

$$ \mathrm{Type}-\mathrm{specific}\ \mathrm{prevalence}\ \left(\mathrm{HPV}\right)=\frac{\mathrm{Number}\ \mathrm{of}\ \mathrm{women}\ \mathrm{HPV}\ \mathrm{positive}\ (n)}{\mathrm{Total}\ \mathrm{number}\ \mathrm{of}\ \mathrm{women}\ \mathrm{tested}\ (N)}\times 100\%. $$

### Data extraction

Data will be extracted into a standardised extraction template and verified independently by a second reviewer using Microsoft Excel™. For study characteristics, the data extracted will include the location of study (city, municipality, prefecture and region), study year (year), study sample type (population based, convenient or others), setting (hospital or clinic), study design (RCT, cohort, case-control or cross-sectional), sample collection method (swab, cytobrush, cervical or vaginal wash or others), sample collection (self-collection, practitioner or others), type of cervical specimen (fresh biopsy, fixed biopsy or exfoliated), cell storage medium, HPV assay, PCR primers used and HPV typing method (DNA or RNA).

In the same extraction template, the sample size of the number of women tested (*N*) and the number of HPV-positive women (*n*) will be extracted. Where available, these will be grouped by cytological (normal, ASCUS, LSIL and HSIL) and histological classification (normal, CIN1, CIN2, CIN3, AIS, ICC—unspecified, ICC—squamous cell carcinoma or ICC—adenocarcinoma).

HPV genotype-specific prevalence data will be extracted and grouped by individual HPV genotypes, presence of any HPV type, any high-risk type (16, 18, 31, 33, 35,39, 45, 51, 52, 56, 58, 59), any low-risk type (6, 11), only low-risk types and genotypes affected by cross-protection (31, 33, 45). In addition, data will also be extracted by vaccine type. This will include the bivalent, quadrivalent and nonavalent vaccines. Each of which will include the corresponding combination of constituent HPV genotypes, i.e. one or more of those types was detected in that specimen. These are bivalent (16 and 18), quadrivalent (6, 11, 16 and 18) and nonavalent (6, 11, 16, 18, 31, 33, 45, 52 and 58), accordingly. Subsequently, genotypes that are defined as probably carcinogenic (68) and possibly carcinogenic (26, 53, 66, 67, 67, 70, 73, 82, 30, 34, 69, 85, 97) will also be extracted and grouped. Finally, prevalence data will also be extracted and grouped by HPV primary screening-detectable genotypes dependent on existing detection methods. For cohort and randomised studies, only the baseline prevalence data will be extracted.

### Managing missing data

In the event that data is missing, the corresponding author of the study will be contacted via email and missing data will be requested. A second email will be sent after 1 week in the event of no response to the initial email. A 2-week waiting period after the submission of the second email will be allowed for sufficient response. After which, these studies will be excluded [31].

### Critical appraisal

The critical appraisal for all included studies will be performed using the ‘Joanna Briggs Institute Prevalence Critical Appraisal Tool’, by two independent reviewers (Table S3) [33]. This critical appraisal tool uses 9 criteria to evaluate studies; a ‘Yes’, ‘No’ response is required for each of the 9 criteria. Where assessment against a criterion is not possible because of incomplete data, then it will be recorded as ‘unclear’ against that particular criterion. Any conflicts that occur between reviewers will be resolved by discussion with a third independent reviewer until consensus is reached.

Studies that meet all quality appraisal criteria are categorised as being high-quality studies. Studies that do not meet 1 or more of the required quality appraisal criteria are categorised as low-quality studies. For studies where a quality appraisal criterion is ‘not applicable’, the reviewers would then discuss the results of the categorisation. If consensus on the final critical appraisal cannot be concluded then, a third reviewer may be required.

### Data analysis and synthesis

Stata version 15 will be used, utilising ‘Metaprop’, a Stata command to perform a meta-analysis of binomial data to calculate pooled prevalence estimates as described below [34]. Analysis will be performed using the Freeman-Tukey double arcsine transformation, and Der Simonian-Laird random-effects methods will be used to compute the weighted overall pooled estimates with confidence intervals (CIs) [35].

Statistical heterogeneity will be quantified using Cochran’s *Q* and the *I*^2^ test statistic to determine the extent of variation in effect estimates that is due to heterogeneity rather than chance. Cochrane’s *χ*^2^ *Q* test statistic will be performed using an *α* cut-off level of 10% [31]. The *I*^2^ test statistic will be used to quantify statistical heterogeneity between studies: heterogeneity from 0 to 30% will be classified as might not be important; heterogeneity from 30 to 75% will be classified as may represent moderate heterogeneity; and heterogeneity from 75 to 100% will be classified as considerable heterogeneity. In this study, the random-effects model will be chosen over the fixed-effects model. If there is substantial heterogeneity above 75%, a meta-analysis will not be performed.

#### HPV genotype-specific prevalence estimates

The pooled genotype-specific HPV prevalence for each HPV genotype or genotype group as previously defined will be estimated independently. Where data allows, pooled estimates will also be stratified by cytological disease stage: normal, ASCUS, LSIL and HSIL, and histological classification is defined as normal, CIN1, CIN2, CIN3, AIS, ICC—unspecified, ICC—squamous cell carcinoma or ICC—adenocarcinoma.

#### Age-specific prevalence estimates

The age-specific prevalence of each HPV genotype or genotype group as previously defined will be calculated for 5 year age groups for the interval 20 to 69 years. Studies that do not report age-specific HPV prevalence will be excluded from age-specific estimates.

#### Vaccine-type prevalence estimates

The proportion of vaccine-preventable infections will be estimated for each vaccine type group as previously defined. Where data allows, pooled estimates will also be calculated and stratified by vaccine type. This will include the bivalent, quadrivalent and nonavalent vaccines.

#### HPV prevalence estimates by geographic location

In order to examine the geographical distribution of HPV genotypes across Japan, pooled estimates of HPV genotype prevalence will be estimated nationally and stratified by region, prefecture and municipality where data allows.

### Assessment and management of heterogeneity

Subgroup analyses and meta-regression will be conducted to identify sources of between-study heterogeneity in the pooled prevalence of HPV infection. Subgroups to be investigated will include study sample type (population based or convenient), study design (cross-sectional or case-control study; RCT or cohort), year of publication, sample collection device, cell storage medium, HPV assay, primers used and HPV typing method. The relative reduction of between-study variance (τ2) will provide an indication of the factor’s contribution to heterogeneity.

### Sensitivity analysis

Sensitivity analysis will be conducted to assess the impact of studies with a low and high critical appraisal score. The impact of specific studies on the pooled prevalence estimate will be determined by exclusion.

### Assessment of reporting bias

The potential for publication or reporting bias will be explored by funnel plots and Egger’s regression asymmetry test, where at least 10 studies are available. Asymmetry of funnel plots will indicate the presence of publication bias [36]. A *p*-value below 10% with Egger’s test will be considered statistically significant.

## Discussion

The outcomes of this study will provide, for the first time, national and subnational estimates of HPV infection in Japan. It is intended that the outcomes of this study will provide evidence in order to evaluate the impact of HPV vaccination to protect against HPV infection and related disease, and inform action needed to eliminate vaccine-preventable cervical cancer in Japan. It will do this by assessing the age distribution of the prevalence of HPV infection, in addition to providing national and regional prevalence estimates of vaccine-preventable and screening-detectable HPV infections from women with cytology- or histology-confirmed normal, low- and high-grade lesions and cervical cancer in Japan.

### Strengths and limitations of this study

- This is the first systematic review protocol that includes both Japanese and English peer-reviewed articles for the determination of genotype-specific prevalence of HPV in normal cervical cytology specimens, low- and high-grade intraepithelial lesions and cervical cancers in Japan.
- This is the first systematic review evaluating the overall prevalence of human papillomavirus stratified by municipality, prefecture or region in Japan.
- This protocol was developed in line with the Preferred Reporting Items for Systematic Reviews and Meta-Analyses guidelines for protocols (PRISMA-P).
- This study only consists of published data or epidemiological studies from female populations participating in cervical HPV sampling or screening programmes.
- A limitation will be that the quality of findings from this review will be dependent on the availability, number and quality of studies included in the final review.

### Ethics and dissemination

Given that the data used in this study will be published and anonymised, publicly available and peer reviewed, ethical approval is not a requirement. This review will be reported in line with the PRISMA statement and will include the PRISMA Checklist. The findings will be published in a peer-reviewed journal and as part of a doctoral thesis.

## Supplementary Information

**Additional file 1: Table S1.** PRISMA-P Checklist for the Japan HPV genotype prevalence systematic review

**Additional file 2: Table S2.** Detailed database Search Strategy for HPV genotype prevalence systematic review for Japan

**Additional file 3: Table S3.** JBI Critical appraisal checklist items for studies reporting prevalence data

## Abbreviations

AIS: Adenocarcinoma in situ
ASCUS: Atypical squamous cells of undetermined significance
CIN: Cervical intraepithelial neoplasia
CIN1: Cervical intraepithelial neoplasia 1
CIN2: Cervical intraepithelial neoplasia 2
CIN3: Cervical intraepithelial neoplasia 3
Covidence: Comprehensive systematic review software (https://www.covidence.org/home)
DNA: Deoxyribonucleic acid
HPV: Human papillomavirus
HSIL: High-grade squamous intraepithelial lesion
ICC: Invasive cervical cancer
LSIL: Low-grade squamous intraepithelial lesion
MHLW: Ministry of Health Labour and Welfare
PROSPERO: International Prospective Register of Systematic Reviews (https://www.crd.york.ac.uk/prospero/)
RNA: Ribonucleic acid
