# Supplementary material for: National genotype prevalence and age distribution of human papillomavirus from infection to cervical cancer in Japanese women: a systematic review and meta-analysis protocol
Source: Syst Rev. 2021 May 5;10:135. doi: 10.1186/s13643-021-01686-6 (PMC8101252; doi:10.1186/s13643-021-01686-6)
Supplement: Supplementary file 2 — Additional file 2: Table S2. Detailed database Search Strategy for HPV genotype prevalence systematic review for Japan [file 13643_2021_1686_MOESM2_ESM.docx]

Table S2: Detailed database Search Strategy for HPV genotype prevalence systematic review for Japan.

| No. | Search Set | Medline/ Pubmed | Embase | Ichushi |
| --- | --- | --- | --- | --- |
| 1 | Population | Japan ti, ab | Japan ti, ab | (日本/TH or 日本/AL) |
| 2 | Population | Japan [Mesh] | Japan ti, ab [Emtree] | NA |
| 3 | Exposure | ‘Human Papillomavirus’ OR ‘HPV’ OR ‘Papillomaviridae’  ti, ab | ‘Human Papillomavirus’ OR ‘HPV’ OR ‘Papillomaviridae’  ti, ab | ((パピローマウイルス科/TH or ヒトパピローマウイルス/AL)) or ((パピローマウイルス科/TH or HPV/AL)) or ((パピローマウイルス科/TH or パピローマウイルス科/AL)) |
| 4 | Exposure | ‘Human Papillomavirus’ OR ‘HPV’ OR ‘Papillomaviridae’, [MeSH] | ‘Human Papillomavirus’ OR ‘HPV’ OR ‘Papillomaviridae’, [Emtree] | NA |
| 5 | Normal | Normal AND Cytology [MeSH] | Normal AND Cytology [Emtree] | (細胞診陰性/AL) or ((細胞診/TH or 細胞診/AL)) |
| 6 | Abnormal | ‘Cervical Cancer’ OR ‘Cervical Disease’ OR ‘Cervical Intraepithelial Neoplasia’ [MeSH] | ‘Cervical Cancer’ OR ‘Cervical Disease’ [Emtree] | ((子宮頸部腫瘍/TH or 子宮頸がん/AL)) or ((子宮頸/TH or 子宮頸部/AL)) or ((子宮頸/TH or 子宮頸部/AL) and (上皮内癌/TH or 上皮内新生物/AL)) |
| 7 | Detection | Genotype [Mesh] | Genotype [Emtree] | (遺伝子型/TH or 遺伝子型/AL) |
| 7 | Complete | #1 AND #3 AND #4 AND #5 AND #6 AND #7 | #1 AND #3 AND #4 AND #5 AND #6 AND #7 | #1 AND #3 AND #5 AND #6 AND #7 |
| 8 | Complete | #2 AND #3 AND #4 AND #5 AND #6 AND #7 | #2 AND #3 AND #4 AND #5 AND #6 AND #7 | NA |
| 9 | Abnormal | #1 AND #3 AND #6 AND #7 | #1 AND #3 AND #6 AND #7 | #1 AND #3 AND #6 AND #7 |
| 10 |  | #2 AND #3 AND #6 AND #7 | #2 AND #3 AND #6 AND #7 | NA |
| 11 | Normal | #1 AND #3 AND #5 AND #7 | #1 AND #3 AND #5 AND #7 | #1 AND #3 AND #5 AND #7 |
| 12 |  | #2 AND #3 AND #5 AND #7 | #2 AND #3 AND #5 AND #7 | NA |
