# Supplementary material for: National genotype prevalence and age distribution of human papillomavirus from infection to cervical cancer in Japanese women: a systematic review and meta-analysis protocol
Source: Syst Rev. 2021 May 5;10:135. doi: 10.1186/s13643-021-01686-6 (PMC8101252; doi:10.1186/s13643-021-01686-6)
Supplement: Supplementary file 3 — Additional file 3: Table S3. JBI Critical appraisal checklist items for studies reporting prevalence data [file 13643_2021_1686_MOESM3_ESM.docx]

Table S3: JBI Critical appraisal checklist items for studies reporting prevalence data

|  | Yes | No | Unclear | Not applicable |
| --- | --- | --- | --- | --- |
| 1. Was the sample frame appropriate to address the target population? | □ | □ | □ | □ |
| 1. Were study participants sampled in an appropriate way? | □ | □ | □ | □ |
| 1. Was the sample size adequate? | □ | □ | □ | □ |
| 1. Were the study subjects and the setting described in detail? | □ | □ | □ | □ |
| 1. Was the data analysis conducted with sufficient coverage of the identified sample? | □ | □ | □ | □ |
| 1. Were valid methods used for the identification of the condition? | □ | □ | □ | □ |
| 1. Was the condition measured in a standard, reliable way for all participants? | □ | □ | □ | □ |
| 1. Was there appropriate statistical analysis? | □ | □ | □ | □ |
| 1. Was the response rate adequate, and if not, was the low response rate managed appropriately? | □ | □ | □ | □ |
